# Lost Dynamics and the Dynamics of Loss: Longitudinal Compression of Brain Signal Variability is Coupled with Declines in Functional Integration and Cognitive Performance

**DOI:** 10.1093/cercor/bhab154

**Published:** 2021-07-23

**Authors:** Douglas D Garrett, Alexander Skowron, Steffen Wiegert, Janne Adolf, Cheryl L Dahle, Ulman Lindenberger, Naftali Raz

**Affiliations:** Max Planck UCL Centre for Computational Psychiatry and Ageing Research, Max Planck Institute for Human Development, Lentzeallee 94, Berlin 14195, Germany; Center for Lifespan Psychology, Max Planck Institute for Human Development, Lentzeallee 94, Berlin 14195, Germany; Max Planck UCL Centre for Computational Psychiatry and Ageing Research, Max Planck Institute for Human Development, Lentzeallee 94, Berlin 14195, Germany; Center for Lifespan Psychology, Max Planck Institute for Human Development, Lentzeallee 94, Berlin 14195, Germany; Max Planck UCL Centre for Computational Psychiatry and Ageing Research, Max Planck Institute for Human Development, Lentzeallee 94, Berlin 14195, Germany; Center for Lifespan Psychology, Max Planck Institute for Human Development, Lentzeallee 94, Berlin 14195, Germany; Research Group of Quantitative Psychology and Individual Differences, Faculty of Psychology and Educational Sciences, KU Leuven, Leuven 3000, Belgium; Institute of Gerontology, Wayne State University, 87 East Ferry Street, Detroit, MI 48202, USA; Max Planck UCL Centre for Computational Psychiatry and Ageing Research, Max Planck Institute for Human Development, Lentzeallee 94, Berlin 14195, Germany; Center for Lifespan Psychology, Max Planck Institute for Human Development, Lentzeallee 94, Berlin 14195, Germany; Center for Lifespan Psychology, Max Planck Institute for Human Development, Lentzeallee 94, Berlin 14195, Germany; Institute of Gerontology, Wayne State University, 87 East Ferry Street, Detroit, MI 48202, USA; Department of Psychology, Wayne State University, 87 East Ferry Street, Detroit, MI 48202, USA

**Keywords:** aging, brain signal variability, cortex, episodic memory, fluid intelligence, longitudinal, MRI, perceptual speed, resting-state, striatum, thalamus

## Abstract

Reduced moment-to-moment blood oxygen level-dependent (BOLD) signal variability has been consistently linked to advanced age and poorer cognitive performance, showing potential as a functional marker of brain aging. To date, however, this promise has rested exclusively on cross-sectional comparisons. In a sample of 74 healthy adults, we provide the first longitudinal evidence linking individual differences in BOLD variability, age, and performance across multiple cognitive domains over an average period of 2.5 years. As expected, those expressing greater loss of BOLD variability also exhibited greater decline in cognition. The fronto-striato-thalamic system emerged as a core neural substrate for these change–change associations. Preservation of signal variability within regions of the fronto-striato-thalamic system also cohered with preservation of functional integration across regions of this system, suggesting that longitudinal maintenance of “local” dynamics may require across-region communication. We therefore propose this neural system as a primary target in future longitudinal studies on the neural substrates of cognitive aging. Given that longitudinal change–change associations between brain and cognition are notoriously difficult to detect, the presence of such an association within a relatively short follow-up period bolsters the promise of brain signal variability as a viable, experimentally sensitive probe for studying individual differences in human cognitive aging.

## Introduction

Typically, researchers conceive of variability as neural “noise,” a nuisance factor that presumably interferes with the efficiency of neural processes. However, growing evidence suggests that various forms of neural variability may prove functionally beneficial for neural systems (Garrett, Samanez-Larkin et al. 2013). In a provocative review several decades ago, Pinneo (1966) posited that ubiquitous neural variability “is not merely noise,” but rather an essential substrate for the stable and functional output of a neural system. Notably, computational modeling suggests that networks formed in the presence of greater noise are more robust to disruption, thus enhancing learning and environmental adaptation, and helping to maintain optimal performance (Basalyga and Salinas 2006; Faisal et al. 2008). More generally, variability may reflect greater neuronal dynamic range, which is vital to the adaptability of neural systems by providing a greater range of potential responses to a greater variety of stimuli. For the past 10 years, we have examined these ideas in the context of human aging, during which system flexibility and adaptability are expected to degrade (Grady and Garrett 2013; Garrett et al. 2013a; Waschke et al. 2021). Indeed, in line with the assumption that greater neural variability may confer functional benefits, we continue to find that greater variability of blood oxygen level-dependent (BOLD) signal is more typical among younger adults and persons with better cognitive performance (Garrett et al. 2010, 2011, 2015, 2017; Grady and Garrett 2013, 2018; Garrett et al. 2013a, 2013b; Burzynska et al. 2015; Nomi et al. 2017). As a result, we and others have argued that brain aging may be reconceptualized as a generalized process of “dynamic compression” that yields increased functional rigidity, thus limiting the system’s adaptability to environmental demands and serving as a potential harbinger of cognitive decline.

However, progress in understanding the role of brain signal variability in cognitive aging has been hampered by the exclusive reliance on cross-sectional designs in previous work. Cross-sectional designs are ill-suited for evaluating neural and cognitive change and individual differences therein (Raz et al. 2005; Nyberg et al. 2010; Lindenberger et al. 2011; Raz and Lindenberger 2011; Lindenberger 2014). Longitudinal investigations have contributed significantly to understanding of age-related change in “structural” brain properties, such as regional volume and cortical thickness (Raz et al. 2005; Fjell et al. 2009), white matter organization (Barrick et al. 2010; Bender et al. 2016) and iron accumulation (Daugherty et al. 2015a). These studies confirm that the estimates of age-related change derived from cross-sectional investigations do not faithfully reflect temporal properties of the brain aging process (Lindenberger et al. 2011; Raz and Lindenberger 2011; Raz 2020). In comparison, the extant longitudinal evidence from functional imaging is very sparse, and when available, is typically limited to analyses of change in functional connectivity and average brain signals (Nyberg et al. 2010; Salami et al. 2014; Pudas et al. 2017). Longitudinal studies demonstrate that in healthy adults, aging-based change–change associations between brain and cognition are subtle and difficult to detect, especially at typically short follow-up periods of several years at most (Raz et al. 2008; Daugherty et al. 2015b; Jäncke et al. 2019). In light of robust and well-replicated cross-sectional associations between signal variability, aging, and cognitive performance (Garrett et al. 2010, 2011, 2015, 2017; Grady and Garrett 2013, 2018; Garrett et al. 2013a; Garrett et al. 2013b; Burzynska et al. 2015; Nomi et al. 2017), it is important to ascertain whether BOLD signal variability can also reveal “change–change” associations between brain and behavior over time. In other words, longitudinal studies of concomitant changes in brain signal variability and cognition are essential for integrating resulting insights into existing theories of cognitive and brain aging.

Using BOLD variability for investigating the coupling between cerebral and cognitive change in healthy aging requires consideration of several fundamental properties of the aging process, such as spatial heterogeneity and heterochronicity of brain changes. Studies of age-related changes in brain structure demonstrate relative sparing of the primary sensory areas, while documenting age-related vulnerability of the tertiary association cortices, neostriatum, and cerebellum, with a particular emphasis on the prefrontal (PFC) regions (Raz and Rodrigue 2006; Grady 2012; Lindenberger 2014). In accord with these findings, one of the rare longitudinal functional magnetic resonance imaging (fMRI) studies undertaken thus far has documented differential decline in PFC activation over time in a sample of healthy adults (Nyberg et al. 2010). Cross-sectional work consistently shows greater BOLD variability in the frontal lobes of younger, higher performing adults (Garrett et al. 2010, 2011, 2015, 2017; Grady and Garrett 2013, 2018; Garrett et al. 2013a; Garrett et al. 2013b; Guitart-Masip et al. 2016; Nomi et al. 2017), but it remains unclear whether localized changes in frontal BOLD variability are associated with changes in cognition. Further, whereas lower BOLD variability in the cortex (including PFC) has often been linked to older age and poorer cognitive performance, the opposite is sometimes true for subcortical regions that are connected to it, such as thalamus and striatum (Garrett et al. 2010, 2011; Samanez-Larkin et al. 2010; Guitart-Masip et al. 2016; Good et al. 2020). In other studies, however, elevated BOLD variability in the subcortical nuclei confers functional benefits (Garrett et al. 2013a; Burzynska et al. 2015; Garrett et al. 2015, 2017; Nomi et al. 2017; Grady and Garrett 2018). Clarifying this apparent discrepancy necessitates examination of cortical versus subcortical characteristics of BOLD variability over time.

Crucially, brain signal dynamics within subcortical regions continue to draw substantial attention in their own right. Recently, efforts have increased toward understanding how “locally measured (within region) variability” may reflect synaptic input. Existing theory suggests that a more disconnected, fractionated biological system should be less dynamic across moments (Pincus 1994), but this had not been shown previously in humans. Beyond work showing that the thalamus is highly important for shaping functional brain dynamics in general (Hwang et al. 2017; Garrett et al. 2018; Shine et al. 2019a, 2019b; Kosciessa et al. 2021), we found recently that higher thalamic signal variability represents perhaps the single most important marker of higher (lower dimensional) system-wide functional integration (Garrett et al. 2018). Similarly, moment-to-moment variability in the neostriatum (caudate and putamen) also appears to be an excellent indicator of the brain’s capacity for functional integration (Garrett et al. 2018). However, as for BOLD variability, longitudinal evidence for functional integration does not yet exist. Importantly, the PFC, the neostriatum, and the thalamus are linked by significant network of reciprocal anatomical connections. Within this fronto-striato-thalamic system, the medial dorsal (MD) nucleus has been proposed as a key node, as it may be critical for integrating broad-scale information within PFC via striatal input through the pallidum (Mitchell and Chakraborty 2013; Mitchell 2015; Pergola et al. 2018) and for regulating plasticity and flexibility of PFC-related cognitive functions (Baxter 2013), perhaps yielding greater moment-to-moment signal variability. As a whole, the fronto-striato-thalamic system may present a useful vehicle for elucidating longitudinal change–change relations between brain signal variability, functional integration, and cognitive aging.

In the current study of healthy adults, we provide initial longitudinal evidence linking individual differences in changes in BOLD variability (at rest) and broad-scale cognitive performance over 2.5 years. We hypothesized that participants exhibiting greater loss of BOLD variability over time would be more likely to decline in cognition, and that this relationship would be particularly salient in the fronto-striato-thalamic system.

## Materials and Methods

### Participants

The data for this study were collected within an ongoing longitudinal investigation in a major metropolitan area of the United States of America (Detroit, MI). Assessments occurred approximately 2.5 years apart [mean = 2.51 years {1.92–3.91 years}]. The participants were community-dwelling volunteers recruited through media advertisements and flyers. They were required to be native English language speakers and have at least a high-school education or an equivalence diploma. Persons who reported a history of cardiovascular, neurological, endocrine, or psychiatric disease, head trauma with a loss of consciousness in excess of 5 min., history of drug or alcohol abuse, or the habit of drinking 3 or more alcoholic drinks per day were excluded, as were persons taking centrally acting medications (e.g., anxiolytics, antidepressants, benzodiazepines, antihistamines). All participants were screened for depression using the Center for Epidemiologic Studies Depression (CES-D) Scale (Radloff 1977) with a cut-off of 15 and were right-handed as indicated by a score above 75% on the Edinburgh Handedness Questionnaire (Oldfield 1971). The participants had a Mini-Mental State Examination (Folstein et al. 1975) score of ≥26.

Participants in the current study came from 2 different subsamples from the larger parent sample. All MRI data were acquired using identical sequences on the same MRI scanner. Subsample 1 underwent the first assessment in 2005 and subsample 2 entered the study in 2011. However, the resting state BOLD (rsBOLD) sequence (of key interest in the present paper) was introduced in 2011. At that time, the third occasion of data collection for subsample 1 was near its end, and for subsample 2, baseline data collection was half-way through. Participants were included in the current analyses if they had 2 consecutive waves of rsBOLD data, regardless of when their own rsBOLD data were first collected (see Supplementary Fig. 1 for a sample flow chart). Thus, for example, a subsample 1 person may have had data available from their third and fourth testing occasions, and a subsample 2 person from their first and second testing occasions. For simplicity in the current paper, we refer to the two available waves of resting-state data as “Time 1” and “Time 2,” regardless of when the data were actually collected. The total sample used in the current analyses comprised *n* = 74 adults (see Supplementary Fig. 1). For both subsamples examined here, identical cognitive test batteries were used. See Supplementary Table 1 for descriptive statistics and statistical comparisons of the two subsamples.

### Data Acquisition

#### MRI Data

Imaging was performed at the MRI Research Center at Wayne State University on a 3-Tesla Siemens Verio (Siemens Medical AG, Erlangen, Germany) full-body magnet with a 12-channel head coil. The MRI scanning session, in addition to other sequences of the longitudinal study not shown here, included a resting state functional and an anatomical scan. For the resting state functional scan, 200 volumes of 43 axial slices were acquired sequential using a T2*-weighted echo-planar sequence with the following parameters: repetition time (TR) = 2500 ms, echo time (TE) = 30 ms, flip angle = 90°, pixel bandwidth = 2298 Hz/pixel, GeneRalized Autocalibrating Partial Parallel Acquisition (GRAPPA) acceleration factor PE = 2, field-of-view = 210 mm, matrix size = 64 × 64, and voxel size = 3.3 × 3.3 × 3.3 mm. Participants were instructed to lie still with their eyes open. For the anatomical scan, a 3D T1-weighted magnetization-prepared rapid gradient-echo sequence was acquired with the following parameters: TR = 1680 ms, TE = 3.51 ms, inversion time = 900 ms, flip angle = 9°, pixel bandwidth = 180 Hz/pixel, GRAPPA acceleration factor PE = 2; field-of-view = 256 mm, matrix size = 384 × 384, and voxel size 0.67 mm × 0.67 mm × 1.34 mm.

### Cognitive Measures

We assessed cognitive performance with a comprehensive test battery comprised of paper-and-pencil and computerized tests spanning 5 cognitive domains: fluid intelligence, crystallized intelligence, episodic memory, working memory (WM), and perceptual speed. The abbreviated description of the tests follows below. For further details, see our previous publications (Raz et al. 1998; Burgmans et al. 2011; Persson et al. 2016).

### Fluid and Crystallized Intelligence

The Cattell Culture Fair Intelligence Test (CFIT, Form 3B; Cattell and Cattell 1973) is known for its sensitivity to aging and is commonly used as a marker of fluid intelligence (Gf) in studies of lifespan development (Rabbitt and Lowe 2000). Each of the 4 subtests (CFIT1–CFIT4) consists of 10–14 items tapping different nonverbal abstract reasoning domains covering a wide range of difficulty, including detecting similarities in designs, sequence completion, and solving nonverbal syllogisms. For each item, the participant must derive the rule required to solve the problem. The number of correctly completed items within the allotted time limits (2.5–4 min per subtest) constitutes a subtest score, whereas the total number correct across subtests serves as the general index of performance. Crystallized intelligence (Gc) was assessed through a multiple-choice vocabulary test (V) derived from the Educational Test Services Kit of Factor-Referenced Tests (Ekstrom et al. 1976).

### Episodic Memory

Memory for names immediate (MNi) and delayed (MNd) recall of the Woodcock–Johnson Psychoeducational Battery–Revised (Woodcock and Johnson 1989) served as two measures of episodic memory. A third measure, spatial recall task was adopted from previous work (Salthouse 1994), with minor modifications. Participants viewed a series of 5 × 5 grids with seven shaded squares on a computer monitor and indicated the placement of these shaded squares on test sheets following the disappearance of each grid.

### Working Memory

WM was assessed via two paper-and-pencil tests, Listening Span (LSPAN) (Salthouse et al. 1989) and Size Judgment Span (Cherry and Park 1993), and two computerized versions of an *n*-back task (Dobbs and Rule 1989; Hultsch et al. 1990). The index of performance in LSPAN was the absolute span, calculated by summing the number of correct items across blocks of trials on which the participant answered all items correctly (Engle et al. 1992). We used the number of errors on 3-back (most difficult) trials of the verbal (NB3Ev) and nonverbal (NB3Env) *n*-back task as additional indices of WM performance.

### Perceptual Speed

Four tests of perceptual speed were administered, two paper-and-pencil and two computerized. The paper-and-pencil tests, Letter Comparison and Pattern Comparison were modified from previously published material (Salthouse and Meinz 1995). The computerized measures were response times for the 1-back trials in the aforementioned verbal (NB1RTv) and nonverbal (NB1RTnv) WM task.

### Data Analysis

#### Estimation of Cognitive Constructs

**Figure 1:**
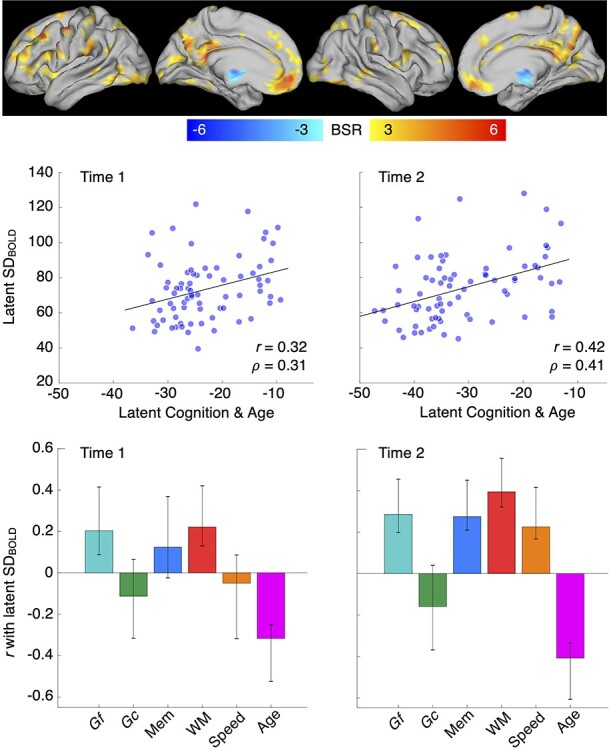
Multivariate model representing cross-sectional relations between SD_BOLD_, cognition, and age. Scatter plots represent the full latent correlation between cognition/age and SD_BOLD_ (shown in Pearson *r* and Spearman’s rho). The bar plots represent how each of the cognitive and age variables contribute to the overall multivariate solution (akin to normalized weights). Confidence intervals in bar plots represent bootstrapped 95% CIs (1000 resamples with replacement).

**Figure 2:**
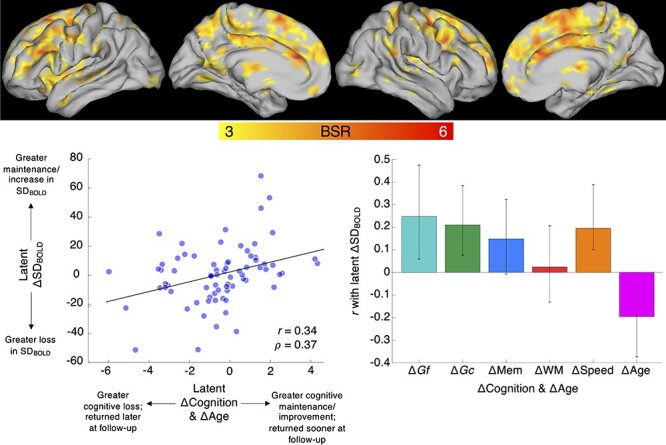
Multivariate model of longitudinal change–change associations between SD_BOLD_, cognition, and age. Confidence intervals in bar plots represent bootstrapped 95% CIs (1000 resamples with replacement). Age change represents length of retest interval, which varied between 1.92 and 3.91 years).

To estimate latent representations of each of the above cognitive domains, we computed principal component scores separately for Time 1 behavioral scores, for Time 2 behavioral scores, and for behavioral change (Time 2 minus Time 1 behavioral difference scores) using SPSS 26 (IBM, Inc.). Only single-component models were estimated for each domain. Standardized loadings for within-domain indicators are shown in Supplementary Table 2. The resulting behavioral component scores were used in the multivariate PLS models reported below in the Results section (Figs 1 and 2).

### Preprocessing of MR Data

The resting-state fMRI data were preprocessed with FSL 5 (RRID:SCR_002823) (Smith et al. 2004; Jenkinson et al. 2012). Preprocessing included motion-correction, smoothing (7 mm kernel), detrending (up to second order) using SPM8, and bandpass filtering (0.01–0.10 Hz; eight order Butterworth filter as implemented in MATLAB version 2014b). Four volumes (10 s) from the resting-state fMRI time series data were discarded prior to preprocessing. We also utilized extended preprocessing steps to further reduce potential data artifacts (Garrett et al. 2010, 2011, 2015; Garrett et al. 2013b). Specifically, we subsequently examined all functional volumes for artifacts via independent component analysis within-run, within-person, as implemented in FSL/MELODIC (Beckmann and Smith 2004). Noise components were identified according to several key criteria: 1) the presence of spiking (components dominated by abrupt time series spikes); 2) motion: prominent edge or “ringing” effects, sometimes, but not always, accompanied by large time series spikes; 3) Susceptibility and flow artifacts: expressed as prominent air-tissue boundary or sinus activation, these typically represent cardio/respiratory effects; 4) activation in the white matter and the ventricles (Birn 2012); 5) low-frequency signal drift (Smith et al. 1999); 6) high power in high frequency ranges unlikely to represent neural activity (≥75% of total spectral power present above 0.10 Hz); and 7) spatial distribution: a “spotty” or “speckled” spatial pattern that appears scattered randomly across ≥25% of the brain, with few if any clusters with ≥25 contiguous voxels at 3.3 × 3.3 × 3.3 mm voxel size.

Examples of these various components we typically deem to be noise can be found in our previous work (Garrett et al. 2013a). By default, we utilized a conservative set of rejection criteria; if manual classification decisions were challenging due to mixing of “signal” and “noise” in a single component, we generally elected to keep such components. Two independent raters of noise components were utilized; > 90% inter-rater reliability was required on separate data before denoising decisions were made on the current data. Components identified as artifacts were then regressed from corresponding fMRI runs using the regfilt command in FSL. Finally, we registered functional images to participant-specific T_1_ images, and from T_1_ to 3-mm standard space (MNI 152_T1) using FLIRT (affine). Prior to registration, T_1_ images were brain extracted with ANTS (version 2.2.0) using a reference template based on the OASIS dataset (Avants and Tutison 2018). The resulting images were masked using the gray matter values provided in FSL (probability > 0.37) and constrained to voxels showing activation across all subjects.

### Voxel-Wise Estimates of Signal Variability

Brain signal variability was calculated using the standard deviation (SD_BOLD_) from the filtered, detrended time series (see above) for each voxel. This quantity is akin to the square root of the total power from the exact same time series (Taylor et al. 2012).

### Statistical Modeling Using Partial Least Squares

To examine multivariate relations between SD_BOLD_ and cognition/age cross-sectionally and longitudinally, we used the multivariate behavioral PLS analysis framework (McIntosh et al. 1996; Krishnan et al. 2011). As the model form is effectively the same when running the cross-sectional model (i.e., cross-sectional data are entered rather than change-based data; see Fig. 1), we will focus on exemplifying the longitudinal model here. This analysis begins by calculating a between-subject correlation matrix (CORR) between voxel-wise change in SD_BOLD_ and cognition and age change. In the next step, the singular value decomposition (SVD) of CORR is computed.

SVD_CORR (delta SDBOLD, delta behavior/age)_ = USV*′* (1)

This decomposition produces a left singular vector of behavior weights (*U*), a right singular vector of brain voxel weights (*V*), and a diagonal matrix of singular values (*S*). Each resulting latent variable (LV) contains a spatial activity pattern depicting the brain regions that show the strongest change–change relation of signal variability to behavior and age identified by the LV. Each voxel weight (an element of *V*) is proportional to the voxel-wise correlation between delta SD_BOLD_ and delta cognition and age.

Significance of detected relations was assessed using 1000 permutations of the singular value corresponding to the LV, followed by bootstrapping with 1000 resamples (Efron and Tibshirani 1993) to evaluate the robustness of the results. By dividing each voxel’s weight (from *V*) by its bootstrapped standard error, we obtained “bootstrap ratios” (BSRs) as normalized estimates of robustness. For the whole brain analysis, we thresholded BSRs at values of ±3.00 [which exceeds a 99.5% confidence interval {CI}].

We also obtained a summary measure of each participant’s expression of a particular LV’s spatial pattern (a within-person “brain score”) by multiplying the model-based vector of voxel weights (*V)* by each subject’s vector of voxel SD_BOLD_ values (*P*), thus producing a single within-subject value,

Brain score = VP*′* (2)

Further, we obtained a summary measure of each participant’s expression of a particular LV’s cognitive/age-based pattern (a within-person “cognition/age score”) by multiplying the model-based vector of cognitive/age weights (*U)* by each subject’s vector of cognitive/age values (*Q*), thus producing a single within-subject value,

Cognition/age score = UQ*′* (3)

### Estimation of Functional Integration

In line with our previous approach (Garrett et al. 2018), we computed functional integration for each subject by running “spatial” principal components analysis (PCA). This method decomposes a correlation matrix for all voxel pairs of interest from each within-subject spatiotemporal matrix),

PCA_voxcorrs_ = USV*´* (4)

where *U* and *V* are the left and right eigenvectors, and *S* is a diagonal matrix of eigenvalues. We then computed a simple, tractable estimate of functional integration by taking the percent variance accounted for by the first principal component (Garrett et al. 2018; Shine et al. 2019a). The larger the variance accounted for by the first principal component for a given subject, the more the voxels correlate with each other across time points in a “unified” manner, and thus, the lower is the system’s dimensionality across time points. PCAs on resting-state data were performed within-subject for each of the 2 testing occasions separately. Change in functional integration was then derived by computing the Time 2 minus Time 1 difference in first component variance accounted for.

### Public Availability

All code necessary to reproduce the current results will appear on Github at https://github.com/LNDG/Garrett_etal_2021_CerebralCortex. Because the MRI and cognitive data are still being collected within an ongoing longitudinal study, they will be made openly available only after completion of the study and dissemination of results.

## Results

### Cross-Sectional Associations between BOLD Signal Variability and Cognitive Performance/Age

We evaluated a single cross-sectional PLS model linking SD_BOLD_ to component scores for each cognitive domain and age at Times 1 and 2. This analysis revealed a single robust LV (permuted *P* < 0.0001) showing that overall, better performing, younger adults expressed higher SD_BOLD_ (Time 1 latent *r* = 0.32; Time 2 latent *r* = 0.42). Such effects were particularly robust at Time 1 for *Gf* and WM and at Time 2 for *Gf*, memory, WM, and speed; see Fig. 1). Age was a strong negative correlate at both Time 1 and 2. Peak effects (see Supplementary Table 3) spanned multiple brain regions. The most prominent positive peaks were evident in canonical default mode regions (precuneus/posterior cingulate and medial PFC), lateral PFC, lateral temporal cortex, and ventral visual cortex. Striatum (especially its ventral region) was also present (see Supplementary Fig. 2), but the thalamus was notably absent in this model. A single, small “negative” peak (representing higher SD_BOLD_ with older age and poorer performance) was also noted, which we discuss in detail in Supplementary Table 3.

**Figure 3:**
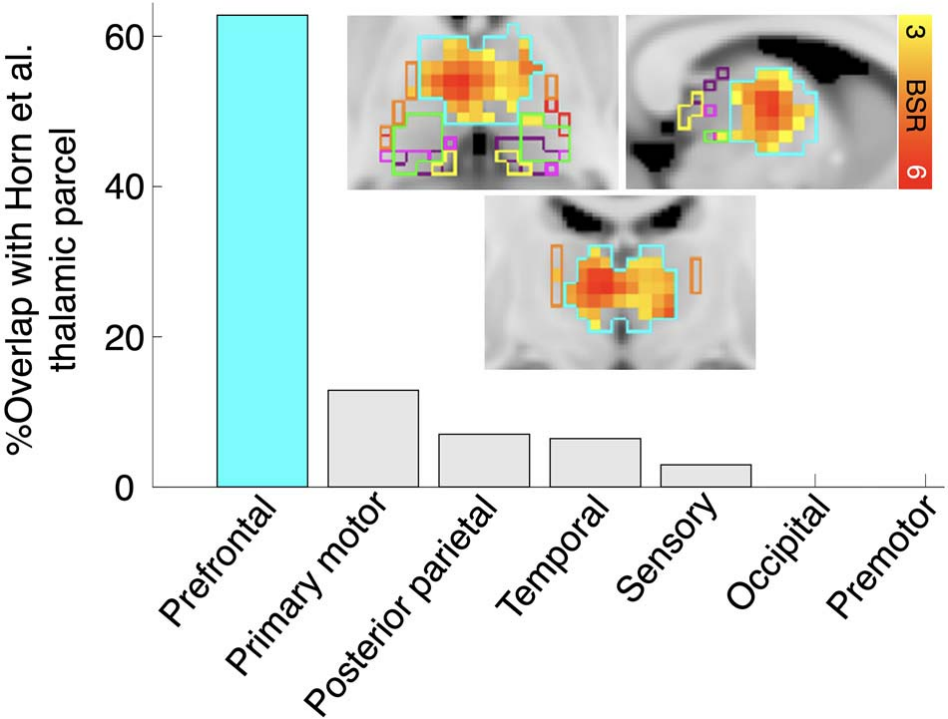
Thalamic change–change effects. Parcels are derived from probabilistic white matter projections from thalamus to cortex (Horn et al., 2016). Following intersection of each thalamic region with the Harvard-Oxford subcortical atlas (Frazier et al. 2005), all bars represent proportions of total voxels within each parcel expressed by above-threshold thalamic voxels within our change–change model. For example, the prefrontal parcel (teal bar and spatial outline) contains 279 voxels, of which 175 were present in our change–change model result (Figure 2 and Supplementary Fig. 3), accounting for 63% coverage. Proportions for other parcels were derived from the following ratios: primary motor (5/39), posterior parietal (7/101), temporal (9/140), sensory (3/102), occipital (0/16), and premotor (0/25).

### Longitudinal Change–Change Relations between SD_BOLD_ and Cognition

Our primary goal in the present study was to examine the relationship between longitudinal changes in SD_BOLD_, cognition, and aging. Under the assumption that cognitive changes may be shared across domains (Tucker-Drob et al. 2019), we fit a unified behavioral PLS model using all change-based cognitive domain-specific component scores to examine coupling between changes in SD_BOLD_ and cognition as well as with advancing age). We found a single robust LV (permuted *P* < 0.0001) revealing that participants who demonstrated greater loss of SD_BOLD_ also exhibited greater decline of cognition, whereas those who maintained (or even increased) BOLD variability maintained (or improved) cognitive performance (latent *r* = 0.34). Bootstrap-stable model weights (i.e., weights for which bootstrap 95% CIs did not contain zero) were evident for *Gf*, *Gc*, and Speed. Peak effects (see Fig. 2, Supplementary Fig. 4, and Supplementary Table 4) spanned a variety of cortical regions, mostly within the frontal cortex, but also in anterior and posterior cingulate gyri, precuneus, and superior parietal lobule.

**Figure 4:**
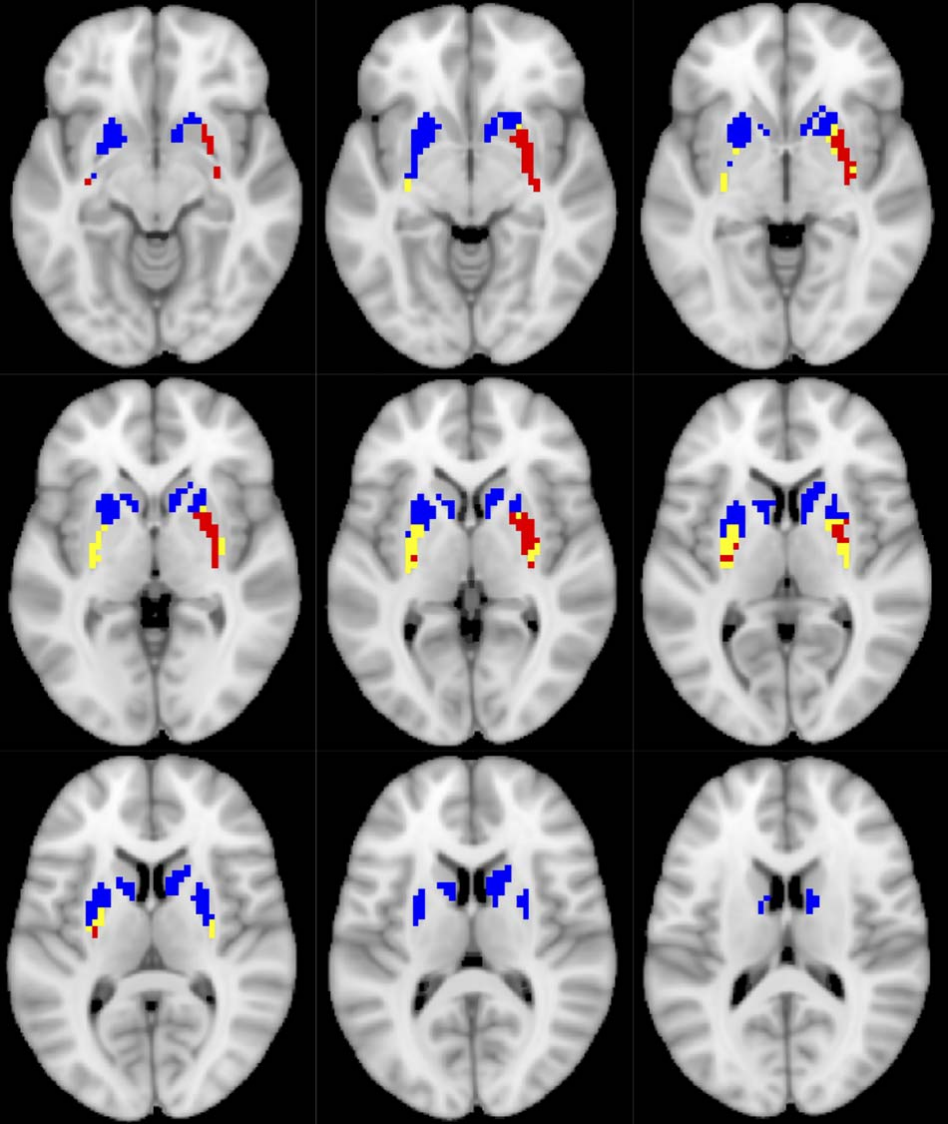
Striatal comparison of cross-sectional (red) and change–change effects (blue), and their overlap (yellow).

An examination of subcortical regions revealed strong bilateral contributions of the thalamus and striatum in our change–change model. We first examine the thalamic effect in greater detail. Given the prominence of the PFC in the overall change–change model (Fig. 2 and Supplementary Fig. 3), it is plausible that thalamic nuclei known for their significant projections to PFC may also feature prominently in the observed effects. Indeed, our findings confirm this expectation. Using the Horn et al. thalamic parcellation, which maps probabilistic white matter connections from thalamus to cortex (Horn and Blankenburg 2016), we observed that in the change–change model, 63% of the thalamic “PFC parcel” was covered by robustly activated voxels (Fig. 3 depicts the dominance of the PFC thalamic parcel within our change–change results). We further characterized the presence of robust thalamic change–change voxels using the Morel histological thalamic atlas (Krauth et al. 2010; Hwang et al. 2017). Convergent with the Horn et al., PFC-centric thalamic parcellation results (Fig. 3), a very large proportion of nucleic coverage by our change–change voxels fell within the MD nucleus (75% coverage; Supplementary Fig. 4). Also, the ventral medial, ventral anterior, and ventral lateral nuclei (sometimes collectively referred to as the “motor thalamus”; O’Reilly et al. 2014; Sieveritz et al. 2018) were very strongly represented as well (Supplementary Fig. 4). Finally, dorsal and ventral striatum (bilateral putamen, caudate, and nucleus accumbens) featured prominently in our change–change results (Fig. 4).

It is possible that the observed change–change association between BOLD variability and cognition *per se* may not imply stable longitudinal change. It is conceivable, for example, that daily variations in SD_BOLD_ and cognition are naturally coupled, and do not represent long-term change. To clarify whether change–change relations in our data truly reflect long-term effects, we leveraged individual differences in retest interval (“age change” in Fig. 2) under the expectation that those who returned at a later interval should be more likely to show loss (range of retest interval = 1.92–3.91 years). Indeed, greater age advancement (i.e., later retest interval) reflected greater loss of SD_BOLD_, with an effect size similar to other robust correlations within our multivariate model (see Fig. 2).

### Change–Change Associations are Not Attributable to Metabolic Risk Estimates

As noted in past work (Ghisletta et al. 2019), metabolic syndrome may reflect a mixture of correlated risk factors that may directly promote the development of atherosclerotic cardiovascular disease (Grundy et al. 2005). Unsurprisingly, advanced age is associated with increase in multiple indicators of metabolic risk, which predict declines in cognition and brain structure and function (Raz and Rodrigue 2006; Raz 2020). In past studies on the population from which our current sample was drawn, we observed moderate effects of metabolic risk on brain shrinkage, white matter diffusivity properties, the regional iron content, and cognitive performance (Swan et al. 1998; Bender et al. 2013, 2016; Daugherty et al. 2015a; Tortelli et al. 2017; Ghisletta et al. 2019). To test whether individual differences in metabolic risk accounted for associations between BOLD variability, cognition, and age in the current study, we first created a single PCA-based “baseline metabolic risk” component (using only Time 1 data) using the following measures: systolic blood pressure, fasting glucose, fasting triglycerides, high-density cholesterol, and waist-to-hip ratio (Ghisletta et al. 2019). We then created a second PCA component representing “change in metabolic risk,” which was derived via the raw longitudinal change scores for each of the indicator measures noted above. See Supplementary Table 2 for loadings for both components (note that only *n* = 68 had complete metabolic risk data across measures). Next, we re-examined the strength of the overall latent correlation noted in Figure 2 (see scatter plot) by regressing SD_BOLD_ change on cognitive/age change while controlling for baseline (the first measurement occasion) and change in metabolic risk. Neither baseline metabolic risk (*P* = 0.21) nor change therein (*P* = 0.48) accounted for significant variance in SD_BOLD_ changes. After further controlling for high leverage outliers in the model (observations with Cook’s distance > 0.058 (i.e., surpassing a Cook’s distance threshold rule of thumb of 4/*n*); *n* = 6 cases held out), baseline (*P* = 0.67) and change in metabolic risk (*P* = 0.65) were even less robust. Thus, metabolic risk does not contribute to change–change associations between SD_BOLD_ and cognition/age in the current sample.

### Comparison of Cross-Sectional and Longitudinal Brain Patterns

**Figure 5:**
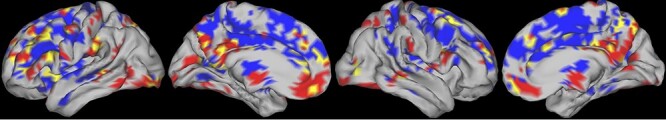
Comparison of cross-sectional (red) and longitudinal (blue) models, and their overlap (yellow).

A direct comparison reveals multiple points of divergence between the cross-sectional and longitudinal models (see Figs 1, 2 and 5, and Supplementary Table 5). The coverage across the frontal lobe appears more extensive across hemispheres in the longitudinal (Fig. 5, blue) than in the cross-sectional model (Fig. 5, red), especially across the bilateral superior midline and left lateral PFC. The cross-sectional model revealed far better coverage of classic default mode nodes (posterior cingulate, ventromedial PFC) and primary and ventral visual cortex. Interestingly, cross-sectional associations in the striatum were a reasonable (although less spatially extensive) proxy for longitudinal change–change coupling (Fig. 4). Putamen and nucleus accumbens showed excellent coverage in both models, although caudate and dorsal putamen was largely apparent only in the longitudinal model. In contrast, despite no discernable cross-sectional effects in thalamus, we observed extensive thalamic coverage in the change–change model (Fig. 3). Overall, overlap between cross-sectional and longitudinal model was modest (Fig. 5, yellow).

**Figure 6:**
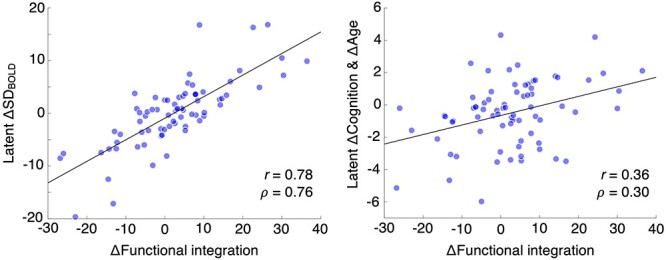
Association between change in functional integration and change in SD_BOLD_ (left panel) and change in cognition and age (right panel). The *y*-axis values here are the same as those represented in Figure 2 (i.e., PLS change–change model results).

### Longitudinal Changes in Functional Integration Track Changes in Cognition and age, Especially among Regions that Express SD_BOLD_ Effects

Previously (Garrett et al. 2018)*,* we showed (in cross-sectional data) that higher local variability (particularly in thalamus, but also in striatum and frontal cortex) covaried strongly with higher functional integration (i.e., “lower dimensionality” of functional connectivity). As such, systems that express higher moment-to-moment variability also have stronger functional connectivity on the same time scale. However, stability of the association between local dynamics and functional integration over time, or association of changes in functional integration and cognitive changes (as already seen for SD_BOLD_ in the current study; see Fig. 2) has not been gauged yet. For these comparisons, we first quantified functional integration among the above-threshold voxels that expressed change–change relations between SD_BOLD_ and cognition, which, as shown before, were dominated by fronto-striato-thalamic regions (see Fig. 2 and Supplementary Fig. 3). Functional integration was operationalized as the percentage of variance accounted for by the first component of a spatial principal component analysis (PCA; see Materials and Methods). PCAs were performed for each time point separately and change in functional integration was derived by computing the Time 2 minus Time 1 difference in variance accounted for. We found that participants who exhibited greater loss in SD_BOLD_ also showed greater decline in functional integration over time (*r* = 0.78, *P* = 2.43 e-16; see Fig. 6, left panel). We also found that loss of functional integration was coupled with a loss of latent-level cognition and older age (i.e., the same latent cognition/age score estimated from our PLS model in Fig. 2; *r* = 0.36, *P* = 0.002; see Fig. 6, right panel). Thus, those who showed greater functional disintegration, mainly in the fronto-striato-thalamic system, also showed more negative change in cognition over time.

Next, we examined the regional specificity of the association between changes in functional integration and changes in cognition and age. In the analysis above, we used the same above-threshold voxels as noted for SD_BOLD_ in the change–change model, so that we could compare SD_BOLD_ to functional integration in the exact same regions when linking them to changes in cognition and age. However, it is possible that the relation between functional integration and cognition/age changes is not regionally specific; if so, it should not matter which voxels we use to compute changes in functional integration. To examine this, we first drew 100 random samples of the same number of voxels (as in the above threshold pattern for SD_BOLD_ noted in Fig. 2; *n* = 10 878 voxels) from all available under-threshold voxels (*n* = 38 981 voxels); doing so ensures that the total estimable dimensionality is equated. The distributions of estimated changes in functional integration from these samples were approximately normal for each subject, and the SDs of these distributions were overall quite small (median SD: 0.15; range: 0.11–.27). This shows that the estimated changes in functional integration for each subject are quite similar across the sampled voxels. We then selected the sample with the smallest cumulative distance to each subject’s estimated median change in functional integration for further analysis. We chose a specific sample rather than the median change in functional integration for each subject to ensure that we compare the effect in the same set of regions across subjects. We found that the relation between change in functional integration and change in latent cognition/age (*r* = 0.25, *P* = 0.04) weakened significantly relative to functional integration change computed from above-threshold voxels (Steiger’s (1980) *Z_diff_* test = 2.48, *P* = 0.007). Given that voxels just below threshold will be included in the random draws prior to estimation of functional integration, it should also be the case that estimating functional integration from voxels further away from threshold should further weaken relations to cognitive change. To estimate this, we selected an equally sized set of voxels set furthest from threshold (which approximately corresponds to the BSR range from 0 to 1.43) and computed change in functional integration in the same way as above. As expected, the correlation with cognitive change weakened further still (*r* = 0.18, *P* = 0.14; Steiger’s *Z_diff_* test = 2.79, *P* = 0.003) relative to when within-threshold voxels are used to compute functional integration. These results suggest that associations between functional integration and cognitive/age change are indeed spatially specific and predominantly driven by the fronto-striato-thalamic system, just as they are for SD_BOLD_.

## Discussion

In this study, we provide first longitudinal evidence for change–change coupling between moment-to-moment BOLD variability, cognition, and aging. In line with past work supporting the utility of brain signal variability for elucidating neural correlates of cognitive aging (Grady and Garrett 2013; Garrett et al. 2013b; McIntosh 2019), we found that persons who showed greater longitudinal reductions in BOLD variability were more likely to evidence degraded cognitive performance over time, whereas their peers who exhibited stable (or increased) BOLD variability also maintained (or improved) cognitive performance. In neuroimaging studies, broad-scale change–change associations between the brain and cognitive variables are strikingly difficult to detect (Raz et al. 2008; Daugherty et al. 2015a; Jäncke et al. 2019). Thus, the robust coupling effects observed here strengthen the proposition that BOLD variability is a viable method for studying individual differences in functional brain and behavioral change in humans.

### Comparison of Cross-Sectional and Longitudinal Models

The extant literature reveals significant discrepancies between cross-sectional and longitudinal models, confirming the limited utility of using cross-sectional age-related differences as proxies for change. This point has been repeatedly made in methodological papers (Lindenberger and Pötter 1998; Hofer and Sliwinski 2001; Lindenberger et al. 2011; Raz and Lindenberger 2011) and illustrated in neuroimaging studies (Raz et al. 2005; Raz 2020). The current study reveals similar discrepancies between cross-sectional and longitudinal models, now within the domain of BOLD signal variability. Our cross-sectional and longitudinal findings converged to demonstrate associations between higher (cross-sectional) and better-maintained (longitudinal) SD_BOLD_ with better (cross-sectional) and better-maintained (longitudinal) cognitive performance. A more detailed comparison, however, reveals diverging patterns of age- and cognition-related associations with BOLD variability across cortical and subcortical regions. In past work, lower cortical variability was nearly uniformly associated with advanced age and poorer cognitive performance (Garrett et al. 2010, 2011; Samanez-Larkin et al. 2010; Guitart-Masip et al. 2016; Good et al. 2020), whereas no consistent pattern has emerged in subcortical nuclei. In some studies, subcortical regions such as striatum and thalamus show the opposite association seen in cortex, with younger, higher cognitive performers exhibiting “lower” SD_BOLD_ (Garrett et al. 2010, 2011; Samanez-Larkin et al. 2010; Guitart-Masip et al. 2016; Good et al. 2020); however, in other studies, signal variability in both cortex and subcortical nuclei exhibit same direction of effect (Garrett et al. 2013b; Burzynska et al. 2015; Garrett et al. 2015, 2017; Nomi et al. 2017; Grady and Garrett 2018). Convergent with the latter set of studies, we found no directional discrepancy between cortical and subcortical regions in either our cross-sectional or longitudinal model in the current study. Higher cortical and subcortical variability was indicative of better cognitive performance, and over time, better-maintained variability was linked to better-maintained cognition. Thus, our study provides and important example of how, despite the general consistency of the direction of effects across cross-sectional and longitudinal models, the results of analyses diverge in many important ways.

### Spatial Patterns

Two key sets of differences between cortical regions in our cross-sectional and longitudinal spatial patterns were present, despite modest overlap. First, classic default model nodes were far more prominent when examining cross-sectional associations. This difference may reflect greater long-term stability within the classic default mode hubs when examined using BOLD variability. It may also indicate that change in DMN signal variability is simply a less sensitive marker of change–change relations with cognition and age over the relatively short follow-up interval (~2 years) implemented in this study. Second, although the current and past (Garrett et al. 2010, 2011, 2015, 2017; Grady and Garrett 2013, 2018; Garrett et al. 2013a; Guitart-Masip et al. 2016) cross-sectional work demonstrates that elevated BOLD variability in the PFC is typical of younger, higher performing adults, the broader fronto-striato-thalamic system became salient only in our change–change model. In particular, thalamic regions projecting directly to the PFC stood out in the evaluation of change–change associations.

In line with the PFC-centric thalamic parcellation results based on Horn and Blankenburg (2016), a very large proportion of nucleic coverage by change–change voxels fell within the MD nucleus. This conforms to the MD’s dense network of projections to and from PFC association areas, and its role as a neural substrate of learning and memory (Mitchell and Chakraborty 2013; Mitchell 2015; Pergola et al. 2018). The MD thalamus has also been proposed as a key node within the generalized fronto-striatal-thalamic circuitry, and as a recipient of striatal input via the pallidum, it may be critical for integrating broad-scale information within PFC (Mitchell and Chakraborty 2013; Mitchell 2015; Pergola et al. 2018). Notably, contrary to being a simple relay to prefrontal cortex, MD may be more specifically involved in the regulation of plasticity and flexibility of PFC-related cognitive functions (Baxter 2013), perhaps yielding greater moment-to-moment signal variability. Second, the ventral medial, ventral anterior, and ventral lateral nuclei were also strongly represented (Supplementary Fig. 4). These nuclei (comprising the so-called “motor thalamus”) connect primarily to premotor, motor, and supplementary motor cortices in the frontal lobe (O’Reilly et al. 2014; Sieveritz et al. 2018), presumably conveying information about movement and movement programs to frontal cortex. Notably, each of these motor-based frontal regions was also present in our change–change model (Fig. 2 and Supplementary Fig. 3). Finally, the presence of various nonfrontal regions in the change–change model suggests that thalamic nuclei projecting to a broader swath of the cortex may also be involved. Indeed, intralaminar (IL) nuclei were well represented in change–change (Supplementary Fig. 4). Previous work suggests that calbindin-positive matrix cells are prominent in the IL and other medial thalamic nuclei (e.g., ventral medial, as in the present results), a cell type that projects diffusely to superficial layers across the neocortex and may even constitute a thalamic “activating system” that drives effective interactions among multiple cortical areas (Honjoh et al. 2018).

Further, the dorsal and ventral striatum (bilateral putamen, caudate, and nucleus accumbens) featured prominently in the observed pattern of change–change coupling (Figs 2 and 4, and Supplementary Fig. 3). These nuclei have been linked to goal-directed-action and motor program execution, to control of motivation and response to reward (Hart et al. 2014), and are vulnerable to aging (Raz et al. 2010; Daugherty et al. 2015b). Importantly, both dorsal and ventral striatum communicate with frontal cortex primarily via the same thalamic nuclei that together dominate the change–change coupling in the thalamus noted above (i.e., MD, ventral lateral, and ventral anterior nuclei) (Hart et al. 2014; O’Reilly et al. 2014). Thus, our results place the fronto-striato-thalamic system at the core of neural substrates of change–change associations between BOLD signal variability, cognition, and aging.

### Cognitive Effects

Although the overall direction of effects was similar in cross-sectional and longitudinal estimates (higher/better-maintained SD_BOLD_ was generally cognitively beneficial), the strength and prominence of cognitive domains differed between models. One clear difference was that WM emerged as a relatively strong positive correlate of SD_BOLD_ in cross-sectional analyses, in line with previous work (Garrett et al. 2011, 2015; Garrett et al. 2013a; Guitart-Masip et al. 2016; Alavash et al. 2018), but showed no robust change–change coupling. Conversely, *Gc* was not associated with SD_BOLD_ in our cross-sectional data, whereas it evidenced a reliable change–change association. As demonstrated by a recent meta-analysis (Tucker-Drob et al. 2019), individual differences in *Gc* changes are common and contribute to a general factor of cognitive change. Overall, we show here that the presence or absence of cross-sectional effects does not necessarily signal how cognition may reflect SD_BOLD_ in change, demonstrating the importance of longitudinal designs (Raz et al. 2005, 2010; Nyberg et al. 2010; Lindenberger et al. 2011; Raz and Lindenberger 2011; Lindenberger 2014) for understanding relations between brain signal variability and cognition.

### Declines in Functional Integration Mirrored Losses in SD_BOLD_ and Cognition

Expanding on previous cross-sectional work (Garrett et al. 2018), we observed first evidence that longitudinal declines in functional network integration (i.e., loss of a lower-dimensional functional network regime) were strongly linked to losses in SD_BOLD_ and to declines in cognition. These effects were especially salient within the same fronto-striato-thalamic regions that typified the SD_BOLD_-based change–change model (Fig. 2). Our findings indicate that adults who were able to maintain a functionally integrated (low dimensional) fronto-striato-thalamic system also succeeded in maintaining higher moment-to-moment brain dynamics and cognition. We argued previously (Garrett et al. 2018) that there are several ways in which higher local variability may emerge in a lower-dimensional (more integrated) functionally connected brain. Computational and animal models suggest that greater moment-to-moment local variability may be driven by networks with balanced excitation and inhibition (E/I), especially when connections are clustered or structured (Shew et al. 2009, 2011; Litwin-Kumar and Doiron 2012; Doiron and Litwin-Kumar 2014; Doiron et al. 2016). “Clustering of connections” is precisely what is captured by higher functional integration (lower dimensional network structure) in the present study. Such E/I balanced networks ensure that fluctuations in synaptic input (via network connectivity) reliably produce output fluctuations at the single-cell level (Shadlen and Newsome 1994; Doiron and Litwin-Kumar 2014). That those who better-maintain high levels of local variability and functional network integration also succeed in maintaining cognition over time suggests that E/I balance may undergird the ability of some persons to maintain cognition in late life. Future longitudinal work using functional magnetic resonance spectroscopy (Stanley and Raz 2018) and/or pharmacological manipulations to probe glutamate and GABA changes may provide a fruitful route for assessing this potential mechanism of cognitive aging.

### Limitations and Future Directions

The current study represents the first foray into establishing change–change relations between brain signal variability, aging, and cognition. Whereas our findings demonstrate the fruitfulness of this approach, several potential limitations need to be addressed for this line of work to progress in the future. First, the current study contained only two testing occasions. A third occasion of data collection is still in progress, and its completion will permit verifying the stability of the observed effects, as well as the evaluation of potential nonlinearities in trajectories of change. Additional measurement occasions will further enable examination of lead–lag effects and discernment of whether loss of brain signal variability precedes cognitive change (or vice versa). It may also be likely that the effect sizes seen in the present change–change model underestimate the true magnitude of the associations that would emerge over a longer follow-up, an expectation supported by our observation that greater compression of SD_BOLD_ was exhibited by participants who returned for their follow-up at a later interval (see Fig. 2). Second, although the current data support a promising initial trait-level description of change–change relations between brain signal variability, cognition, and aging, longitudinal “task-based” fMRI data remain required to tap into cognitive processes more directly, and to more specifically probe the nature of the fronto-striato-thalamic system dynamics that are so prominent in our results.

Finally, although the causality of the observed effects remains unclear, evidence suggests that longitudinal brain changes (in structure or function) can co-occur with changes in brain vasculature and/or cardiovascular health. In the current sample, we found no impact on change–change associations between SD_BOLD_ and cognition/age after controlling for both baseline and change in metabolic risk. Despite this lack of association with extra-experimental risk factors, only assessment of the vascular factors concurrent with acquisition of the BOLD signal can elucidate the impact of age-related changes in vascular reactivity and other cerebrovascular properties. Such assessment likely requires the collection of carbogen-based hypercapnia data, which were unavailable in the current study. Although we have found previously that such regional control of aging-related vascular differences does not fully eliminate cross-sectional age differences in SD_BOLD_ (Garrett et al. 2017), it remains unknown whether longitudinal hypercapnia-based controls might reduce change-based associations between SD_BOLD_ and cognition to a greater or lesser extent than cross-sectional associations. Although useful and interesting in their own right (Tsvetanov et al. 2015, 2021; Millar et al. 2020), externally measured vascular and metabolic risk factors that affect cerebral vasculature and associated responses are insufficient for resolving these issues, especially when cross-sectional comparisons and mediation models are used (Nyberg et al. 2010; Lindenberger et al. 2011; Raz and Lindenberger 2011). Direct measures of vascular contribution from the same brain regions for which SD_BOLD_ is estimated are absolutely necessary for inference about local vascular versus neural variability effects (Gauthier and Hoge 2012; Gauthier et al. 2013; Liu et al. 2013; Golestani et al. 2015, 2016). ASL-based cerebral blood flow estimates are relatively straightforward to acquire, but such data account for a negligible proportion of variance in SD_BOLD_ (Garrett et al. 2017), suggesting that a carbogen-based hypercapnia approach likely remains required for a thorough examination of these important issues.

### Summary

We found that healthy adults who lost moment-to-moment BOLD signal variability over a 2.5-year period also declined in cognition and functional integration, whereas those who maintained/increased variability also maintained/increased their levels of cognitive performance and functional integration. The examination of BOLD variability thus represents a sensitive and viable approach for understanding neural and behavioral changes during adulthood and old age. In particular, the fronto-striato-thalamic system emerged as a core neural substrate for these change–change associations and may present an important target in future investigations of the dynamic functional neural bases of human cognitive aging.

## Funding

Emmy Noether Programme grant from the German Research Foundation (to D.D.G.); International Max Planck Research School (IMPRS) LIFE and IMPRS COMP2PSYCH PhD programs (A.S.); Intramural Innovation Fund of the Max Planck Society (to U.L.). Max Planck UCL Centre for Computational Psychiatry and Aging Research (D.D.G. and U.L.); National Institutes of Health, United States of America (grant R01/R37 AG011230 to N.R.).

## Notes

We would like to thank the editor and 3 anonymous reviewers for their helpful suggestions on our manuscript. *Conflict of Interest*: We have no conflicts of interest to report.

## Supplementary Material

GarrettEtAl-DetroitRsVariabChangechange-Supp-FINAL_bhab154
