# Supplementary material for: Lost Dynamics and the Dynamics of Loss: Longitudinal Compression of Brain Signal Variability is Coupled with Declines in Functional Integration and Cognitive Performance
Source: Cereb Cortex. 2021 Jul 23;31(11):5239–52. doi: 10.1093/cercor/bhab154 (PMC8491679; doi:10.1093/cercor/bhab154)
Supplement: GarrettEtAl-DetroitRsVariabChangechange-Supp-FINAL_bhab154 [file garrettetal-detroitrsvariabchangechange-supp-final_bhab154.docx]

|  | Total Sample = 74 | | Sub-sample 1 = 22 | | Sub-sample 2 = 52 | | Sub-sample comparison | | |
| --- | --- | --- | --- | --- | --- | --- | --- | --- | --- |
|  | Time 1 | Time 2 | Time 1 | Time 2 | Time 1 | Time 2 | Statistic | Value | *p* |
| % Female | 58.11 | n/a | 68.18 | n/a | 53.85 | n/a | χ^2^ | 1.31 | 0.25 |
| % Caucasian | 79.73 | n/a | 81.82 | n/a | 78.85 | n/a |  | 0.08 | 0.77 |
| % Exercisers | 82.43 | 85.14 | 90.91 | 81.82 | 78.85 | 86.54 |  | 1.55 | 0.21 |
| % Non-Smokers | 91.89 | 91.89 | 86.36 | 86.36 | 94.23 | 94.23 |  | 1.28 | 0.26 |
| % Normotensive | 82.43 | 78.38 | 72.73 | 63.64 | 86.54 | 84.62 |  | 1.00 | 0.15 |
| Age (Years) | 51.51 ± 17.23 | 54.02 ± 17.27 | 60.71 ± 11.78 | 63.15 ± 11.84 | 47.62 ± 17.77 | 50.15 ± 17.83 | *t* | 3.17 | 0.002* |
|  | (19.75-82.75) | (22.33-84.75) | (35.33-82.75) | (37.58-84.75) | (19.75-77.83) | (22.33-81.5) |  |  |  |
| Education (Years) | 15.93 ± 1.93 | 16.27 ± 2.00 | 16.14 ± 2.44 | 16.14 ± 2.44 | 15.85 ± 1.69 | 16.33 ± 1.81 |  | 0.59 | 0.56 |
|  | (12-20) | (12-21) | (12-20) | (12-20) | (12-20) | (12-21) |  |  |  |
| MMSE | 28.82 ± 1.04 | 29.04 ± 0.88 | 28.86 ± 1.04 | 29.09 ± 0.81 | 28.81 ± 1.05 | 29.02 ± 0.92 |  | 0.21 | 0.83 |
|  | (26-30) | (27-30) | (27-30) | (27-30) | (26-30) | (27-30) |  |  |  |
| Exercise Frequency (Days) | 3.26 ± 2.20 | 3.98 ± 2.51 | 3.80 ± 2.23 | 3.05 ± 2.17 | 3.04 ± 2.17 | 4.38 ± 2.55 |  | 1.36 | 0.18 |
|  | (0-7) | (0-7) | (0-7) | (0-7) | (0-7) | (0-7) |  |  |  |
| Systolic (mmHg) | 120.35 ± 12.96 | 119.69 ± 13.78 | 122.10 ± 12.46 | 123.48 ± 14.92 | 119.60 ± 13.21 | 118.09 ± 13.08 |  | 0.76 | 0.45 |
|  | (96.5-163.75) | (93.25-160) | (98-153.33) | (93.25-160) | (96.5-163.75) | (93.75-150) |  |  |  |
| Diastolic (mmHg) | 75.87 ± 9.00 | 74.20 ± 7.94 | 75.56 ± 7.42 | 75.18 ± 5.87 | 76.00 ± 9.65 | 73.79 ± 8.69 |  | -0.19 | 0.85 |
|  | (62.5-122.5) | (59.5-101) | (67.33-101.33) | (62-83.75) | (62.5-122.5) | (59.5-101) |  |  |  |
| Glucose (mg/dL) | 88.53 ± 11.32 | 90.04 ± 13.05 | 90.91 ± 9.34 | 93.55 ± 16.17 | 87.52 ± 12.00 | 88.50 ± 11.26 |  | 1.18 | 0.24 |
|  | (66-129) | (68-142) | (78-115) | (77-142) | (66-129) | (68-124) |  |  |  |
| Total Cholesterol (mg/dL) | 181.73 ± 36.37 | 180.86 ± 33.87 | 189.64 ± 36.91 | 190.64 ± 39.36 | 178.39 ± 35.98 | 176.56 ± 30.60 |  | 1.22 | 0.23 |
|  | (104-279) | (113-260) | (137-279) | (119-260) | (104-264) | (113-251) |  |  |  |
| HDL (mg/dL) | 56.92 ± 16.76 | 59.40 ± 15.53 | 59.05 ± 14.43 | 59.64 ± 13.71 | 56.02 ± 17.71 | 59.30 ± 16.39 |  | 0.71 | 0.48 |
|  | (22-98) | (31-93) | (22-88) | (37-90) | (29-98) | (31-93) |  |  |  |
| LDL (mg/dL) | 103.77 ± 29.36 | 101.54 ± 30.30 | 107.05 ± 32.40 | 108.00 ± 37.40 | 102.39 ± 28.20 | 98.70 ± 26.53 |  | 0.62 | 0.54 |
|  | (52-182) | (46-179) | (56-182) | (46-179) | (52-181) | (54-160) |  |  |  |
| Triglycerides (mg/dL) | 105.49 ± 62.95 | 99.76 ± 50.92 | 118.23 ± 66.91 | 115.36 ± 52.86 | 100.10 ± 61.06 | 92.90 ± 49.01 |  | 1.14 | 0.26 |
|  | (24-317) | (31-249) | (42-307) | (42-249) | (24-317) | (31-240) |  |  |  |
| Cholesterol Ratio | 3.44 ± 1.11 | 3.22 ± 0.94 | 3.43 ± 1.20 | 3.34 ± 1.06 | 3.44 ± 1.08 | 3.17 ± 0.88 |  | -0.04 | 0.97 |
|  | (1.9-7.5) | (1.8-7.0) | (2.0-7.5) | (1.8-7.0) | (1.9-6.2) | (1.8-5.4) |  |  |  |
| Waist-to-Hip Ratio (inches) | 0.89 ± 0.09 | 0.89 ± 0.07 | 0.90 ± 0.08 | 0.90 ± 0.07 | 0.89 ± 0.09 | 0.89 ± 0.07 |  | 0.40 | 0.69 |
|  | (0.69-1.09) | (0.72-1.01) | (0.74-1.09) | (0.78-1.0) | (0.69-1.05) | (0.72-1.01) |  |  |  |
| Body Mass Index | 26.16 ± 5.05 | 26.37 ± 4.92 | 26.05 ± 4.84 | 26.33 ± 4.41 | 26.20 ± 5.18 | 26.39 ± 5.16 |  | -0.12 | 0.91 |
|  | (17.41-42.08) | (18.66-38.92) | (17.41-36.96) | (19.48-26.33) | (18.12-42.08) | (18.66-38.92) |  |  |  |

*Table S1: Sample descriptors and comparisons between the two sub-samples.* Proportions or Means +- SDs are reported for each variable, and value ranges for each variable are in brackets. Statistical comparisons were made on Time 1 data for each sub-sample. *Only a single variable (age) significantly differed.

|  |  | Principal component loadings | | |
| --- | --- | --- | --- | --- |
| Cognitive domain | Measure | Time 1 | Time 2 | Change |
| *Gf* | CIFIT 1 | 0.80 | 0.84 | -0.46 |
|  | CIFIT 2 | 0.81 | 0.78 | 0.70 |
|  | CIFIT 3 | 0.71 | 0.65 | 0.83 |
|  | CIFIT 4 | 0.76 | 0.70 | -0.05 |
| Memory | Names (immediate) | 0.94 | 0.95 | 0.84 |
|  | Names (delayed) | 0.94 | 0.93 | 0.87 |
|  | Spatial recall | 0.64 | 0.78 | 0.46 |
| Working memory | Listening span | 0.80 | 0.77 | 0.54 |
|  | Size judgement span | 0.77 | 0.82 | 0.71 |
|  | 3-back errors (verbal) | -0.80 | -0.78 | -0.59 |
|  | 3-back errors (nonverbal) | -0.85 | -0.85 | -0.63 |
| Speed | Letter comparison | 0.76 | 0.79 | 0.78 |
|  | Pattern comparison | 0.75 | 0.86 | 0.46 |
|  | 1-back RT (verbal) | -0.72 | -0.72 | -0.56 |
|  | 1-back RT (nonverbal) | -0.79 | -0.81 | -0.60 |
| Metabolic risk | Systolic blood pressure | 0.69 | - | 0.29 |
|  | Fasting glucose | 0.73 | - | 0.69 |
|  | Fasting triglycerides | 0.70 | - | 0.64 |
|  | High-density cholesterol | -0.65 | - | -0.49 |
|  | Waist-to-hip ratio | 0.47 | - | 0.51 |

*Table S2: Cognitive domain PCA loadings for cross-sectional and change-based models.* Note that no model for crystallized intelligence was run due to the availability of only a single indicator (vocabulary; see methods). All loadings are standardized, representing the Pearson correlation between the indicator and the within-domain latent score (-1 to 1). Note that the moderate negative loading for CIFIT 1 in the *Gf* change model is (unexpectedly) moderately negative. To ensure this outlying indicator did not influence our results, we re-ran the change-change PLS model noted in Figure 3 and found no ostensible change in spatial pattern, strength of the overall latent pattern, or strength of *Gf* itself in the multivariate solution (data not shown).

|  |  | MNI | | |  | Cluster size |
| --- | --- | --- | --- | --- | --- | --- |
| Anatomical region | Hem | X | Y | Z | BSR | (voxels) |
| PCC* | - | 0 | -42 | 18 | 7.93 | 1729 |
| Precentral Gyrus | L | -54 | 12 | 33 | 7.11 | 908 |
| Inferior Temporal Gyrus | L | -54 | -63 | -9 | 5.99 | 410 |
| SupraMarginal Gyrus | L | -66 | -24 | 21 | 5.76 | 599 |
| Superior Medial Gyrus | L | 0 | 39 | 30 | 5.66 | 1165 |
| Middle Frontal Gyrus | L | -30 | 3 | 63 | 5.46 | 174 |
| Pallidum | R | 30 | -12 | -3 | 5.43 | 216 |
| Superior Frontal Gyrus | R | 30 | 60 | 12 | 5.39 | 177 |
| Superior Frontal Gyrus | R | 30 | 6 | 63 | 5.24 | 129 |
| Inferior Parietal Lobule | R | 54 | -30 | 57 | 5.18 | 125 |
| Superior Temporal Gyrus | R | 48 | -30 | -3 | 5.02 | 140 |
| SupraMarginal Gyrus | R | 63 | -24 | 30 | 5.00 | 65 |
| IFG (p. Opercularis) | R | 48 | 15 | 18 | 4.88 | 101 |
| Middle Occipital Gyrus | R | 30 | -66 | 33 | 4.85 | 54 |
| Fusiform Gyrus | R | 42 | -72 | -18 | 4.84 | 287 |
| Superior Temporal Gyrus | R | 54 | 0 | 3 | 4.82 | 30 |
| Lobule I IV** | - | 12 | -30 | -24 | 4.56 | 45 |
| Putamen | L | -27 | -9 | 6 | 4.43 | 74 |
| Temporal Pole | L | -48 | 18 | -12 | 4.22 | 94 |
| Inferior Occipital Gyrus | R | 33 | -93 | -9 | 4.22 | 25 |
| Middle Occipital Gyrus | R | 42 | -81 | 6 | 4.13 | 47 |
| Mammillary bodies*** | - | 0 | -12 | 0 | -5.05 | 76 |

*Table S3: Peaks from cross-sectional PLS model between SD_BOLD_ and behavior.* MNI coordinates, peak bootstrap ratio (BSR) values, and cluster sizes are listed. Anatomical labels generated by the Anatomy Toolbox for SPM. Asterisks denote peaks not defined in SPM Anatomy toolbox: *denotes a large bilateral PCC cluster. **a cluster that spans into Lobule I IV upon manual inspection. ***denotes a cluster which spanned the mamillary bodies and a large portion of the third ventricle (see Figure S2). It is normally the case that our probabilistic grey matter masking step eliminates most ventricles; however, the third ventricle is very proximal to dense grey matter, making it difficult to mask without losing substantial surrounding grey matter tissues. To ensure that our multivariate PLS result (Figure 1, main paper) are not driven by this single negatively signed cluster, we subsequently re-ran a PLS model after masking this cluster out. Results were unchanged; regenerating the latent correlations for Time 1 and 2 from this new model yielded *r_Time1_* = .32 and *r_Time2_* = .42, which are *identical* to values in the original model (see Figure 1, main paper).

|  |  | MNI | | |  | Cluster size |
| --- | --- | --- | --- | --- | --- | --- |
| Anatomical region | Hem | X | Y | Z | BSR | (voxels) |
| Middle Frontal Gyrus* | R | 21 | -3 | 51 | 7.55 | 8270 |
| Superior Parietal Lobule | R | 39 | -63 | 57 | 6.72 | 259 |
| Putamen | R | 18 | 12 | 3 | 6.14 | 1103 |
| Cerebellum (VI) | R | 36 | -57 | -30 | 5.70 | 164 |
| Cuneus | R | 21 | -60 | 21 | 4.97 | 431 |
| Hippocampus* | R | 18 | -21 | -15 | 4.92 | 33 |
| Middle Temporal Gyrus | R | 57 | -15 | -12 | 4.90 | 67 |
| Precentral Gyrus | L | -24 | -21 | 72 | 4.76 | 305 |
| Paracentral Lobule* | - | 0 | -30 | 75 | 4.60 | 36 |
| Middle Temporal Gyrus | L | -57 | 3 | -27 | 4.45 | 57 |
| Area hOc4lp | R | 45 | -87 | 3 | 3.98 | 35 |
| Lingual Gyrus | L | -12 | -66 | -3 | 3.91 | 62 |
| Middle Occipital Gyrus | L | -36 | -93 | -3 | 3.85 | 56 |

*Table S4: Peaks from longitudinal (change-change) PLS model between SD_BOLD_ and behavior.* MNI coordinates, peak bootstrap ratio (BSR) values, and cluster sizes are listed. Anatomical labels generated by the Anatomy Toolbox for SPM. ***denotes peaks not defined in SPM Anatomy toolbox, but which clearly span into the associated regions upon manual inspection (see Figure S3 for full axial view).

*
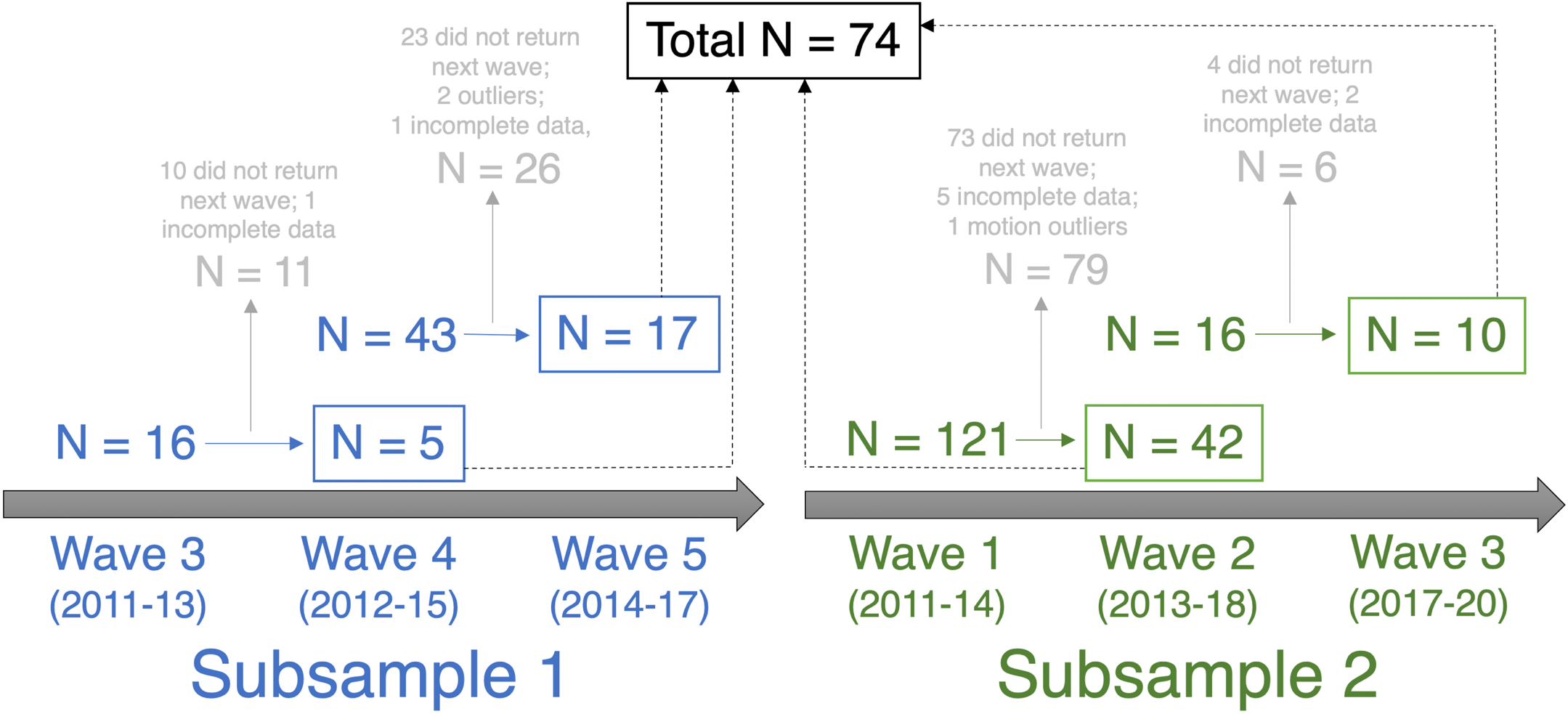
*

*Figure S1: Breakdown of subsamples utilized to arrive at the current sample of n = 74.* The two outliers noted for subsample 1 were due to extremely late retest intervals (>5 years). The two motion outliers in subsample 2 exhibited > 4mm mean absolute displacement. In the current paper, all participants had two consecutive testing occasions available with resting-state BOLD data. For the purposes of simplicity in the present study, the two testing occasions for each participant were then referred to as “Time 1” and “Time 2,” regardless of when the data were actually collected.


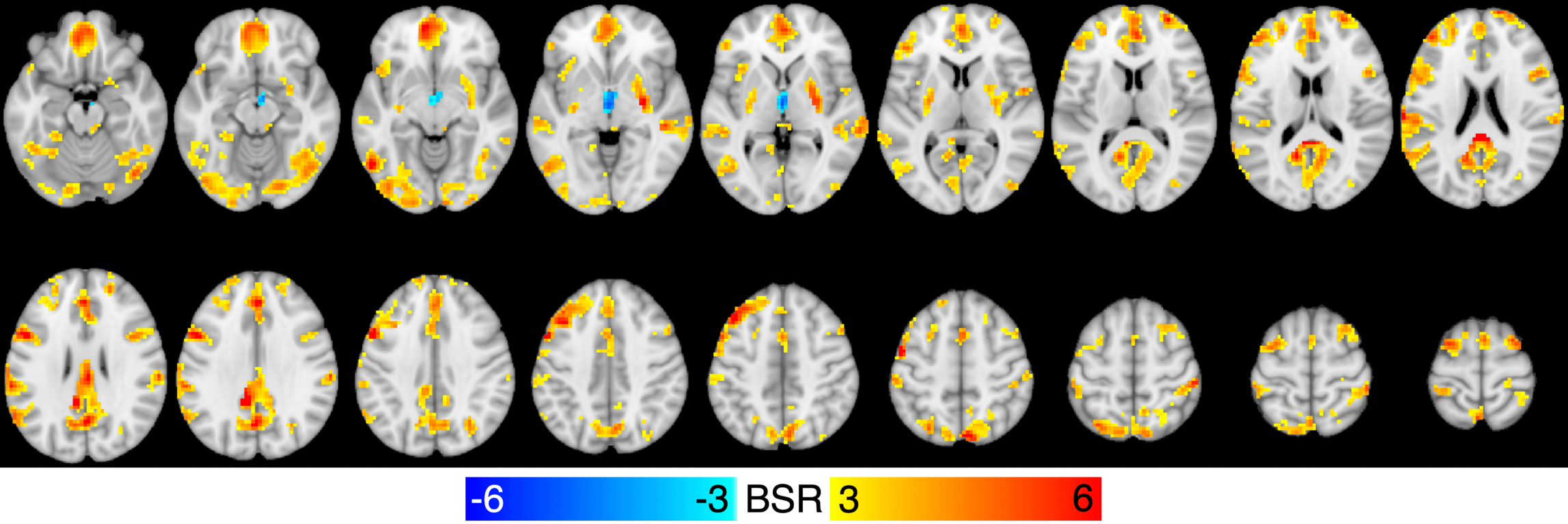


*Figure S2: Axial plot for cross-sectional behavioral PLS model between brain and behavior (see Figure 1).* BSR = bootstrap ratio.


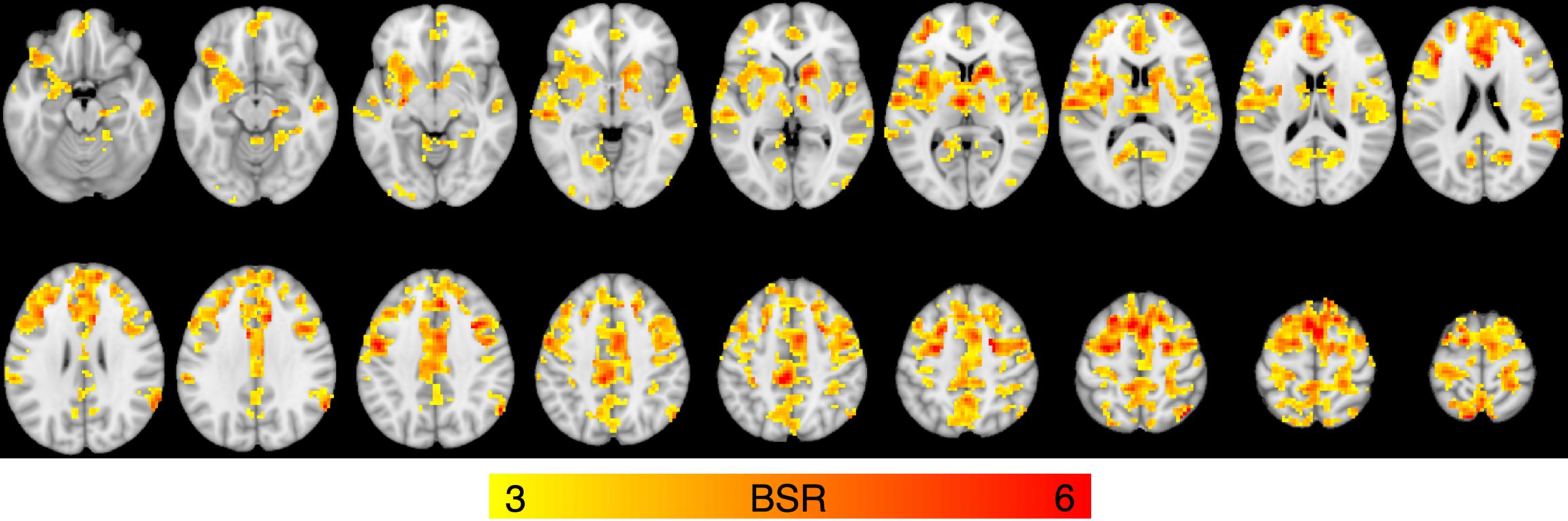


*Figure S3: Axial plot for longitudinal (change-change) PLS model between brain and behavior (see Figure 3).* BSR = bootstrap ratio.

**
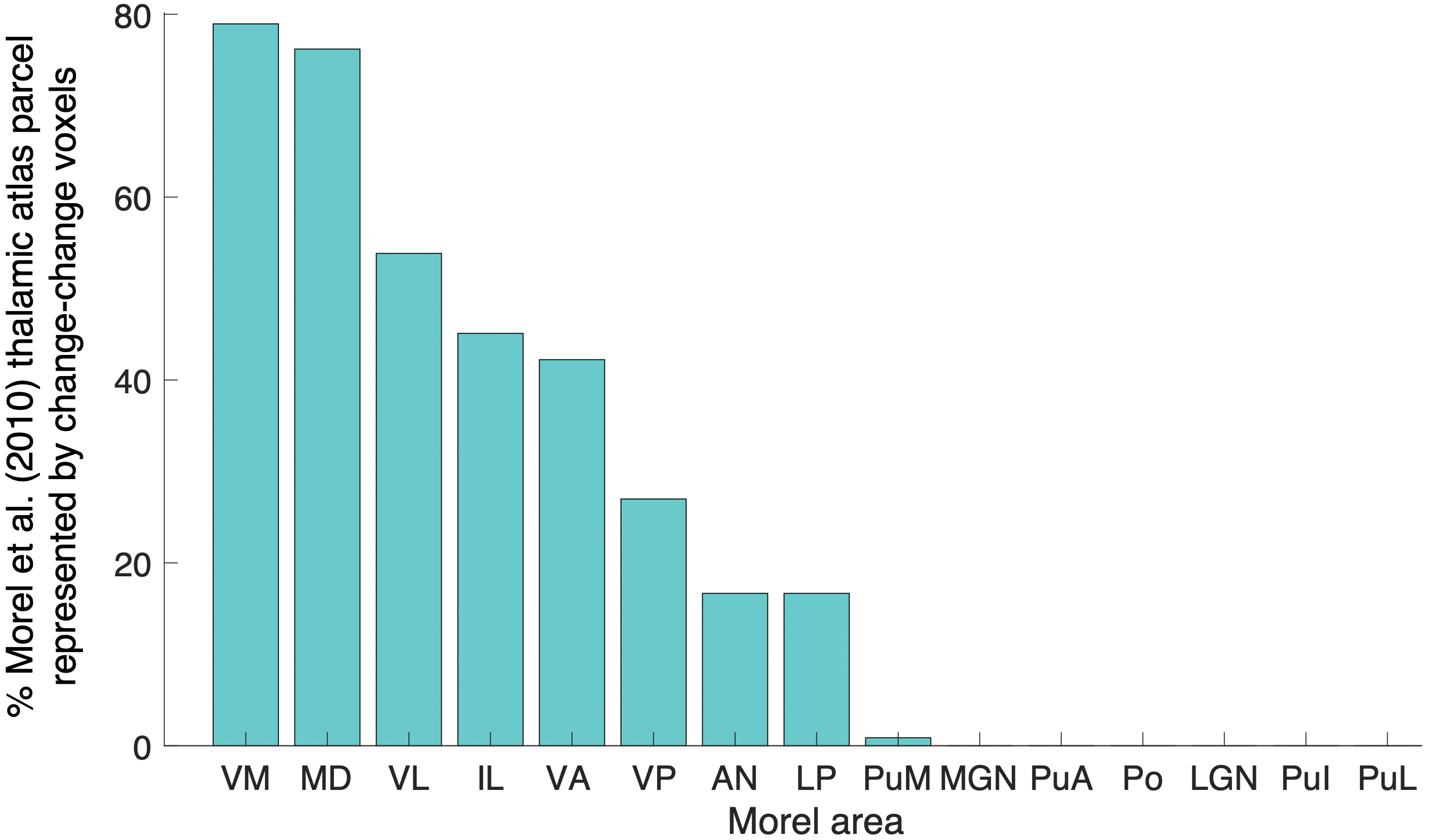
**

*Figure S4: Morel (Krauth et al., 2010) atlas thalamic nuclei represented within multivariate change-change model.* Bars represent proportions of total voxels within each nucleus expressed by thalamic voxels within our change-change model. For example, the MD nucleus contains 63 voxels (3mm isotropic), and 47 of those 63 voxels were present in our change-change model (Figure 3 and S3), accounting for 75% coverage of the MD. Other proportions were as follows: VM (15/19); VA (22/45); VL (66/117); IL (46/102); VP (20/63); LP (5/36); AN (5/24); PuA (0/11); PuM (1/115); MGN (0/13); Po (0/6); LGN (0/12); Pul (0/1); PuL (0/32).


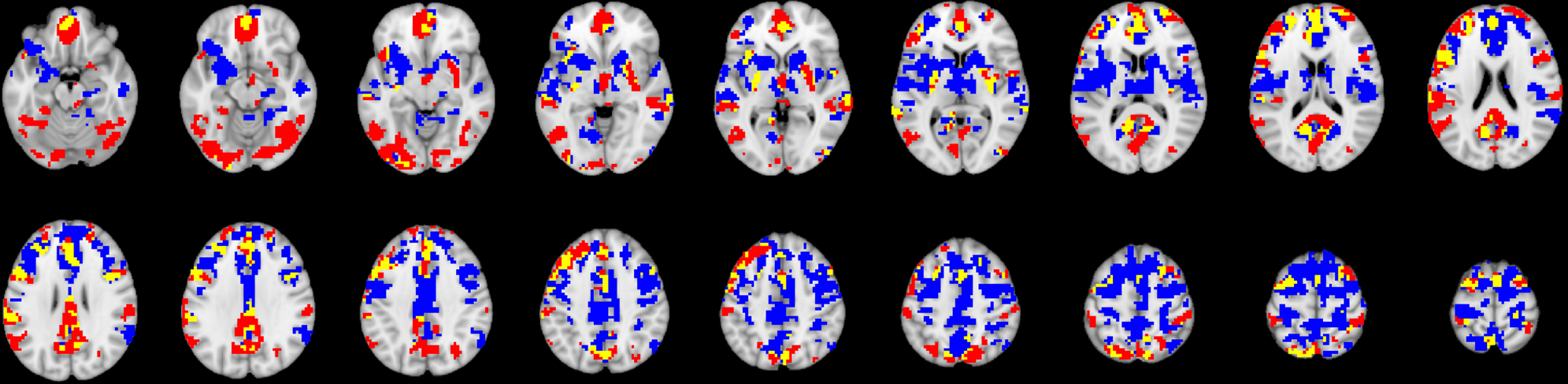


*Figure S5: Axial plot for comparison of cross-sectional (red) and longitudinal effects (blue), and their overlap (yellow).* See Figure 5 for surface plot.
